# 0.33*g* mitigates muscle atrophy while 0.67*g* preserves muscle function and myofiber type composition in mice during spaceflight

**DOI:** 10.1126/sciadv.aed2258

**Published:** 2026-03-13

**Authors:** Ryosuke Tsuji, Ryo Fujita, Takuto Hayashi, Shunya Sadaki, Tatsuya Matsumoto, Yuri Inoue, Yuka Murakami, Michito Hamada, Masafumi Muratani, Hiroe Kobayashi, Akane Yumoto, Maki Okada, Daisuke Kamimura, Risa Okada, Takafumi Suzuki, Ryo Kurosawa, Akihito Otsuki, Seizo Koshiba, Martha Hotz Vitaterna, Charles A. Fuller, Marie Mortreux, Dong-Min Sung, Jason Ciola, Seward B. Rutkove, Jennifer Coulombe, Takashi Kudo, Masayuki Yamamoto, Mary L. Bouxsein, Dai Shiba, Satoru Takahashi

**Affiliations:** ^1^ Laboratory Animal Resource Center in Transborder Medical Research Center, and Department of Anatomy and Embryology, Institute of Medicine, University of Tsukuba, Ibaraki 305-8575, Japan.; ^2^ Division of Regenerative Medicine, Transborder Medical Research Center, Institute of Medicine, University of Tsukuba Ibaraki, Ibaraki 305-8575, Japan.; ^3^ Université Paris Cité, INSERM U1151, CNRS UMR8253, Institut Necker Enfants Malades (INEM), 75015 Paris, France.; ^4^ Department of Genome Biology, Transborder Medical Research Center, Institute of Medicine, University of Tsukuba, Ibaraki 305-8575, Japan.; ^5^ Space Environment Utilization Center, Human Spaceflight Technology Directorate, Japan Aerospace Exploration Agency (JAXA), Ibaraki 305-8505, Japan.; ^6^ Tohoku Medical Megabank Organization, Tohoku University, Sendai, Japan.; ^7^ Advanced Research Center for Innovations in Next-GEneration Medicine (INGEM), Tohoku University, Sendai, Japan.; ^8^ Department of Neurobiology, Northwestern University, Evanston, IL, USA.; ^9^ Department of Neurobiology, Physiology and Behavior, University of California, Davis, CA, USA.; ^10^ University of Rhode Island, Kingston, RI, USA.; ^11^ Beth Israel Deaconess Medical Center, Department of Neurology, Harvard Medical School, Boston, MA, USA.; ^12^ Brigham and Women’s Hospital, Boston, MA, USA.; ^13^ Center for Advanced Orthopedic Studies, Beth Israel Deaconess Medical Center and Department of Orthopedic Surgery, Harvard Medical School, Boston, MA, USA.

## Abstract

As human space exploration advances, understanding how different gravity levels affect skeletal muscle is critical for long-term health. Among the major organ systems, skeletal muscle is particularly sensitive to gravitational unloading, yet the gravity threshold required to maintain homeostasis remains unclear. Using the Multiple Artificial-gravity Research System aboard the International Space Station, mice were exposed to graded gravity levels, microgravity, 0.33*g*, 0.67*g*, and 1*g*, and their muscles were analyzed postflight. In the gravity-sensitive soleus, the cross-sectional area was preserved at 0.33*g*, while the slow-to-fast myofiber transition was partially suppressed at 0.33*g* and fully prevented at 0.67*g*. Functional measures, including forelimb grip strength and electrical impedance myography, indicated that 0.67*g* was sufficient to maintain muscle performance. Plasma metabolomics identified 11 metabolites with gravity-dependent changes, suggesting potential biomarkers for monitoring physiological adaptation. Collectively, these results identify 0.67*g* as a critical threshold for mitigating spaceflight-induced muscle atrophy and myofiber type transitions.

## Teaser

Spaceflight tests reveal that 0.33*g* mitigates muscle loss, but 0.67*g* is needed to keep function, impacting future Mars missions.

## INTRODUCTION

Gravity exerts widespread effects on biological systems, including the cardiovascular, immune, vestibular, and musculoskeletal systems of humans and other organisms (*1*–*4*). In particular, skeletal muscles are overly sensitive to gravity loss, which can result in substantial muscle weakness, muscle atrophy, and myofiber type transition (*5*–*7*). Changes in myofiber types are often accompanied by metabolic alterations in skeletal muscles (*8*). With NASA’s goal of sending humans to Mars in the 2030s, a comprehensive understanding of the molecular mechanisms underlying these gravity-induced changes is urgently required, as is the development of appropriate countermeasures to prevent deleterious effects on skeletal muscles. However, the mechanisms by which gravity regulates skeletal muscle homeostasis and the effects of its absence during spaceflight remain largely unclear, owing to the lack of tools available to study the effect of gravity on mammalian skeletal muscles.

Skeletal muscle comprises distinct myofibers defined by myosin heavy chain isoforms and metabolic activity. Slow-twitch myofibers (type I), expressing myosin heavy chain 7 (*Myh7*), rely on oxidative metabolism for energy production and have high respiratory potential. Fast-twitch myofibers (type II) can be subdivided into three groups: type IIa, type IIx, and type IIb, which are encoded by *Myh2*, *Myh1*, and *Myh4*, respectively (*9*, *10*). Similar to type I, type IIa myofibers mainly use oxidative metabolism, while type IIx and IIb myofibers predominantly rely on glycolytic metabolism. During spaceflight, because of a conversion of slow-to-fast myofibers induced by microgravity (μG), the proportion of fast myofibers increases in the soleus muscle (SOL) (*6*, *11*). While the molecular regulation of myofiber type determination has not been fully elucidated, we previously identified the large Maf transcription factors, comprising *Mafa*, *Mafb*, and *Maf*, as being the principal regulators of type IIb myofiber determination (*8*). The absence of all three large Mafs in the SOL completely prevents the slow-to-fast myofiber transition induced by hindlimb unloading, a model of μG (*8*). Alternately, the overexpression of each large Maf induces fast muscle gene programs that include *MYH4* expression in both mice (*8*) and humans (*12*).

Type I and IIa myofibers are more vulnerable to inactivity and μG than type IIx and IIb (*6*, *11*). Decreased muscle mass and myofiber size are typically caused by an imbalance between protein synthesis and degradation in skeletal muscles. Protein synthesis is promoted through activation of the phosphatidylinositol 3-kinase (PI3K)/Akt/mammalian target of rapamycin (mTOR) signaling pathway, which also down-regulates protein degradation pathways though phosphorylation of muscle atrophy–related genes (atrogenes) (*13*). Protein degradation is mediated by several atrogenes, which are components of the ubiquitin-proteasome and autophagy-lysosome systems (*14*, *15*). The two muscle-specific ubiquitin ligases identified to play a role in muscle atrophy are Atrogin-1/MAFbx and MuRF1/Trim63 (*14*, *16*, *17*). However, the targets of these ubiquitin ligases do not account for the degradation of all muscle proteins during muscle atrophy (*15*). In regard to spaceflight, it has been shown that the deletion of Trim63 does not fully attenuate μG-induced muscle atrophy (*18*). This shows that the canonical Trim63-dependent proteolytic pathway is not the primary driver of muscle atrophy in μG, suggesting that additional or distinct catabolic mechanisms may be activated under μG. Furthermore, suppression of protein synthesis has also been implicated in muscle atrophy under μG (*19*). Together, these findings indicate that, in addition to classical atrogene pathways, other molecular mechanisms may be involved in muscle loss during μG exposure. Although we have previously identified the large Maf transcription factor family as a principal regulator of type IIb myofiber determination, the upstream regulatory networks governing muscle atrophy and myofiber type transition under μG, particularly the fundamental mechanisms of gravity sensing in skeletal muscle, remain largely unknown. Such mechanisms can only be fully elucidated through spaceflight experiments, as μG is a unique environment that cannot be precisely replicated on Earth.

In addition to muscle tissue analysis, circulating factors, such as metabolites and cell-free (cf) DNA/RNA, offer a promising avenue for assessing physiological changes during spaceflight, as they can be measured noninvasively and may reflect systemic adaptations, including those in skeletal muscle. Previous μG studies, including our own, have reported alterations in circulating cfDNA/RNA related to an early frailty phenotype (*20*, *21*). However, the gravity dependence of these changes, as well as their potential utility as biomarkers, has not been fully investigated. While cfDNA/RNA primarily reflects genomic or transcriptional states, metabolites represent the end products of cellular biochemical activity, providing a more direct snapshot of functional metabolic status.

We previously developed the Multiple Artificial-gravity Research System (MARS) on the International Space Station (ISS) (*22*). This system enables the generation of a wide range of gravity levels, from μG to hypergravity, in the space environment, thereby facilitating the study of skeletal muscle responses under different gravitational conditions. On Earth, tail suspension has been widely used as a ground-based analog of spaceflight unloading; however, this model not only unloads hindlimb skeletal muscles but also induces systemic stress due to restraint and altered posture. In contrast, MARS allows the creation of an artificial 1*g* environment in space, where mice are exposed to the same space-specific stressors (e.g., radiation, oxidative stress, and vibration) as those in spaceflight, while only gravity is matched to Earth conditions. This unique feature makes it possible to isolate and analyze the pure effects of gravity on skeletal muscle, which cannot be fully disentangled using ground-based control experiments. Using MARS, we previously investigated skeletal muscle responses to Earth’s gravity (1*g*) and lunar gravity (0.16*g*) on the ISS. We found that while 1*g* completely prevented both muscle atrophy and the slow-to-fast myofiber type transition of the SOL induced by μG (*7*), lunar gravity only prevented muscle atrophy (*6*). These findings suggest that different gravity thresholds exist for regulating muscle mass and myofiber types. If such thresholds exist, the mechanisms by which skeletal muscles sense gravity and convert mechanical signals into biochemical signals remain largely unknown. Understanding these processes is crucial, not only for developing effective countermeasures to protect astronaut health during space missions but also for preventing age- or disease-associated muscle weakness here on Earth, such as from sarcopenia or cachexia. In parallel, identifying circulating metabolites that change in a gravity-dependent manner could provide noninvasive biomarkers for monitoring skeletal muscle health in space and on Earth, offering a valuable tool for both astronaut health surveillance and clinical assessment of muscle-related disorders.

Therefore, our study aimed to determine gravity thresholds capable of mitigating spaceflight-induced alterations in skeletal muscle, including changes in muscle mass, myofiber type composition, muscle function, and gene expression. We hypothesized that these deteriorative processes are governed by gravity-dose–dependent mechanisms, such that partial gravity levels between μG and 1*g* might serve as threshold levels at which muscle degradation can be attenuated. To test this hypothesis, mice were exposed to graded gravity levels (μG, 0.33*g*, 0.67*g*, and 1*g*) for 27 to 28 days using the MARS system aboard the ISS.

## RESULTS

### Influence of gravity variations in spaceflight on skeletal muscle function and mass

**Fig. 1.**
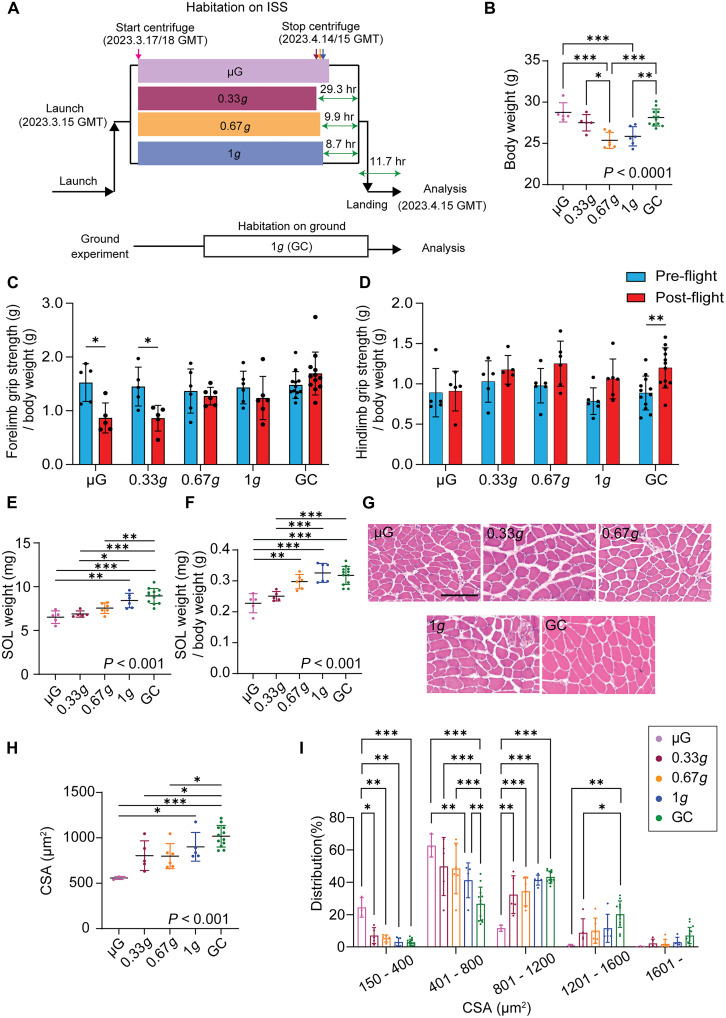
Gravity-dependent changes in the SOL mass during spaceflight. (**A**) Overview of the MHU-8 mission. The duration of exposure to artificial gravity was 27.0 days for the 0.33*g* group, 27.0 days for the 0.67*g* group, and 28.0 days for the 1*g* group. Periods of microgravity (μG) exposure after centrifuge termination are shown in green: 29.3 hours (hr) for the 0.33*g* group, 9.9 hours for the 0.67*g* group, and 8.7 hours for the 1*g* group. All flight groups were subsequently exposed to 1*g* for 11.7 hours after landing. (**B**) Body weight of each group postflight. *P < 0.05, **P < 0.01, and ***P < 0.001, as determined using Tukey’s test. (**C**) Changes in forelimb grip strength from pre- to postflight normalized by body weight. *P < 0.05 (paired *t* tests with Sidák correction for multiple comparisons). (**D**) Changes in hindlimbs grip strength from pre- to postflight normalized by body weight. **P < 0.01 (paired *t* tests with Sidák correction for multiple comparisons). (**E**) SOL weight. *P < 0.05, **P < 0.01, and ***P < 0.001, as determined using Tukey’s test. (**F**) SOL weight normalized by body weight. *P < 0.05, **P < 0.01, and ***P < 0.001, as determined using Tukey’s test. (**G**) H&E staining of the SOL cross sections. Scale bar, 100 μm. (**H**) Cross-sectional area (CSA) of the SOL myofibers. *P < 0.05 and ***P < 0.001, as determined using Tukey’s test. (**I**) CSA distribution of the SOL myofibers. *P < 0.05, **P < 0.01, and ***P < 0.001, as determined using Tukey’s test. Data are shown as mean ± SD. Sample sizes: μG (*n* = 5), 0.33*g* (*n* = 5), 0.67*g* (*n* = 6), 1*g* (*n* = 6), and GC (*n* = 12), unless otherwise noted. For panels analyzed using one-way ANOVA, the *P* value testing the overall effect of gravity is shown within the graph. Post hoc pairwise comparisons are indicated by asterisks.

Twenty-four male C57BL/6J mice were launched to the ISS, where they were exposed to μG, 0.33*g*, 0.67*g*, or 1*g* using the MARS centrifuge system (Fig. 1A). Of the 24 mice, one mouse in the μG group died during the mission, and 23 returned alive. Dissection began approximately 6 hours after landing, representing a substantial improvement over the 2-day delay in our previous study (*2*).

Upon returning from space, the body weights of the mice exposed to 0.67*g* and 1*g* were significantly lower than those in the μG and habitat ground control (GC) groups (Fig. 1B). Postflight, the μG and GC groups exhibited a significant increase in body weight compared with preflight body weight, whereas the 0.67*g* and 1*g* groups exhibited a significant decrease (fig. S1, A to E). There was one mouse in the 0.33*g* group that lost over 10% of its body weight, likely due to water nozzle malfunction. Therefore, that mouse was excluded from our analysis.

To investigate changes in muscle performance after spaceflight under different gravitational conditions, we compared the average grip strength of the forelimbs and hindlimbs before and after flight (Fig. 1, C and D). Forelimb grip strength, normalized to body weight, showed a significant decline after spaceflight in both the μG and 0.33*g* groups compared with preflight levels (Fig. 1C). In contrast, no significant changes were observed in the 0.67*g*, 1*g*, or GC groups, suggesting that higher gravitational loading during spaceflight helps prevent functional decline in forelimb strength. For the hindlimbs, grip strength normalized to body weight did not show significant changes between pre- and postflight in any of the spaceflight groups (Fig. 1D). However, in the GC group, a slight but significant increase in hindlimbs grip strength was observed between baseline and end point measurements.

The SOL, which is highly susceptible to gravitational changes, exhibited significant atrophy in the in the μG and 0.33*g* groups compared with that in the 1*g* and GC groups following spaceflight, both in terms of absolute wet weight (Fig. 1E) and normalized weight (Fig. 1F). The 0.67*g* group showed reduced absolute SOL weight compared with that of the 1*g* group, but this difference was not significant (Fig. 1E). When normalized, the 0.67*g* group did not differ significantly from the 1*g* and GC groups (Fig. 1F).

To further support these findings, we next examined cross sections of the SOL using hematoxylin and eosin (H&E) staining (Fig. 1G). Histological analyses confirmed the absence of abnormal myofiber structures, such as central nucleation, in all groups. Consistent with the reduction in wet weight, the average cross-sectional area (CSA) of the SOL myofibers was significantly decreased in the μG group compared with that in the 1*g* and GC groups (Fig. 1H). In contrast, the 0.33*g* and 0.67*g* groups showed a slight but nonsignificant reduction in average CSA compared with that shown by the 1*g* group. Furthermore, the percentage of myofibers with a CSA ranging from 150 to 400 μm^2^ was significantly higher in the μG group than in any of the other groups, suggesting that 0.33*g* loading during spaceflight would be sufficient to partially mitigate muscle atrophy.

We also investigated the weight and histological characteristics of other hindlimb muscles, including the plantaris, gastrocnemius (GAS), tibialis anterior, and extensor digitorum longus (EDL) muscles (fig. S2, A to D). These muscles are predominantly composed of fast-twitch myofiber types (type IIx/IIb). Although the normalized EDL weight was not reduced in the μG group compared with that in the 1*g* and GC groups (fig. S2D), the normalized muscle weights of the plantaris (at μG), GAS (at μG and 0.33*g*), and tibialis anterior (at μG) were significantly reduced compared with those in the 1*g* and GC groups (fig. S2, A to C). To confirm these observations, we analyzed the CSA of the EDL. The cross-sectional analysis of the EDL did not reveal any differences between the groups (fig. S2, E to G). Collectively, our findings indicated that the regulation of skeletal muscle mass and function exhibited distinct gravity-dependent thresholds. While 0.33*g* was sufficient to mitigate muscle atrophy in the SOL, a higher gravity level, 0.67*g*, was required to maintain muscle performance as measured by grip strength. These results suggest that the gravitational load required to preserve muscle function is higher than that needed to prevent muscle mass loss.

### Influence of gravity variations in spaceflight on myofiber composition and electrical integrity

μG-induced SOL muscle loss was partially attenuated in the 0.33*g* group (Fig. 1). However, the preservation of muscle function, as assessed by grip strength, required higher gravitational loading. To investigate the underlying mechanisms contributing to this discrepancy, we next evaluated intrinsic muscle properties through electrical impedance myography (EIM) and investigated myofiber type composition using immunohistochemical analysis.

**Fig. 2.**
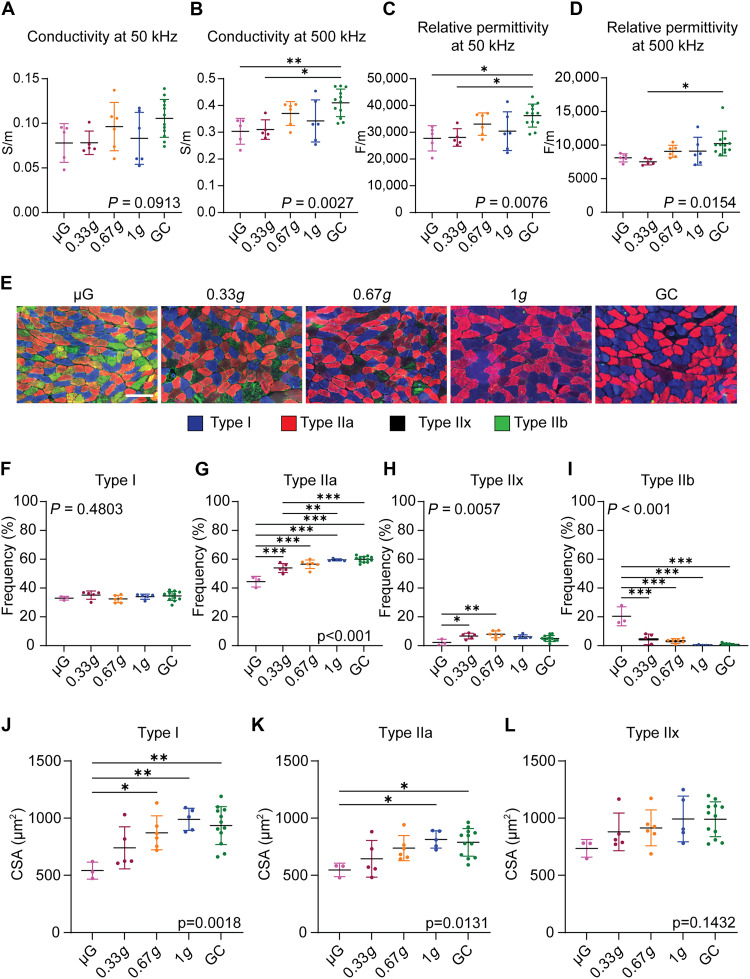
Gravity-dependent changes in myofiber type transition in the SOL during spaceflight. (**A**) Conductivity at 50 kHz. (**B**) Conductivity at 500 kHz. *P < 0.05 and **P < 0.01, as determined using Tukey’s test. (**C**) Relative permittivity at 50 kHz. *P < 0.05, as determined using Tukey’s test. (**D**) Relative permittivity at 500 kHz. *P < 0.05, as determined using Tukey’s test. (**E**) Immunohistochemical staining of myosin heavy chain using BA-D5 (type I; blue), SC-71 (type IIa; red), and BF-F3 (type IIb; green) antibodies. Unstained myofibers are defined as type IIx (black). Scale bar, 100 μm. (**F** to **I**) Frequency of type I/IIa/IIx/IIb myofibers in the SOL under different gravity conditions. *P < 0.05, **P < 0.01, and ***P < 0.001, as determined using Tukey’s test. (**J** to **L**) CSA of distinct myofiber types (type I/IIa/IIx myofibers) of the SOL myofibers. *P < 0.05 and **P < 0.01, as determined using Tukey’s test. Data are shown as mean ± SD. Sample sizes: μG (*n* = 5), 0.33*g* (*n* = 5), 0.67*g* (*n* = 6), 1*g* (*n* = 6), and GC (*n* = 12) for (A) to (D); μG (*n* = 3), 0.33*g* (*n* = 5), 0.67*g* (*n* = 6), 1*g* (*n* = 5), and GC (*n* = 12) for panels (F) to (L). For each quantitative panel, the *P* value from the main statistical analysis (one-way ANOVA testing the overall effect of gravity level) is shown within the graph. Post hoc pairwise comparisons are indicated by asterisks.

We performed EIM measurements at two frequencies (50 and 500 kHz) to assess intrinsic muscle properties using the excised GAS (Fig. 2, A to D). EIM parameters were determined by two physical properties: conductivity, which reflects how easily electrical current passes through skeletal muscle, and permittivity, which reflects the capacity of skeletal muscle to store electromagnetic energy. The conductivity at 500 kHz, but not at 50 kHz, exhibited a significant decrease in the μG and 0.33*g* groups compared with that in the GC group (Fig. 2, A and B). The relative permittivity decreased at both frequencies as gravity levels decreased. The relative permittivity at 50 kHz was significantly lower in the μG and 0.33*g* groups than in the GC group (Fig. 2C). In addition, relative permittivity at 500 kHz was significantly lower in the 0.33*g* group than in the GC group (Fig. 2D). These results suggest that the reductions in relative permittivity and conductivity observed under the μG and 0.33*g* conditions were consistent with the degree of atrophy in the SOL (Fig. 1F) and GAS (fig. S2B), supporting the notion that muscle atrophy is associated with altered intrinsic electrical properties.

To further investigate the intrinsic changes after spaceflight, we examined the myofiber types in the SOL. Exposure to μG induces a slow-to-fast myofiber type transition in the SOL. To assess myofiber type composition, cross sections of the SOL were subjected to immunohistochemical staining with antibodies specific to type I, IIa, and IIb myosin heavy chains (Fig. 2, E to I). Myofibers negative for all three markers were classified as type IIx. Consistent with previous observations (*8*), the proportion of type IIa myofibers in the SOL was significantly reduced under μG compared with that under 1*g* (Fig. 2G). A similar reduction in type IIa myofibers was observed in the 0.33*g* group, but not in the 0.67*g* group, indicating that 0.33*g* is insufficient to prevent the slow-to-fast myofiber type transition in the SOL. Type IIb myofibers, normally scarce in the adult SOL under standard gravity, were significantly increased in the μG group compared with that in the 1*g* group (Fig. 2I). In contrast, mice exposed to 0.33*g* did not show an increase in type IIb myofibers (Fig. 2I). Rather, they exhibited a distinct shift characterized by a decrease in type IIa myofibers (Fig. 2G) along with a corresponding increase in type IIx myofibers (Fig. 2H). Given that myofiber type transition typically proceeds sequentially (type IIa ⇔ IIx ⇔ IIb), these findings implied that under μG, the type IIa myofibers in the SOL were fully converted to type IIb, whereas under 0.33*g* conditions, this transition was arrested at the IIx stage. This suggests that the extent of myofiber type specification is modulated in a gravity reduction-dependent manner and that partial gravity at 0.33*g* cannot fully prevent the reduction of type IIa myofibers in the SOL. In contrast, exposure to 0.67*g* effectively preserved the proportion of type IIa myofibers and prevented the ectopic appearance of type IIb myofibers in this predominantly slow-twitch muscle during spaceflight.

Similar immunohistochemical staining was also performed on the EDL muscle, which predominantly consists of type IIx and IIb myofibers. The results showed a significant increase in the proportion of type IIa myofibers in the μG group compared with that in all other gravity groups (fig. S3, A and B). Concurrently, the proportion of type IIb myofibers was significantly reduced in the μG group relative to that in the 0.67*g*, 1*g*, and GC groups, but not when compared with that in the 0.33*g* group (fig. S3, A and D). These findings indicated that while 0.33*g* was insufficient to attenuate the myofiber type transition in EDL muscle, attenuation was observed at 0.67*g* and higher gravity levels.

We next sought to determine which myofiber type is most susceptible to atrophy during spaceflight (Fig. 2, J to L). Although the CSA of type IIa and IIx myofibers in the SOL showed gravity-dependent changes (Fig. 2, K and L), type I myofibers exhibited the most pronounced CSA reduction in the μG group compared with that in the 0.67*g*, 1*g*, and GC groups (Fig. 2J). Notably, the CSA of type I myofibers in the 0.33*g* group was not significantly different from that in the 1*g* and GC groups (Fig. 2J), indicating that type I myofiber atrophy was partially prevented at 0.33*g*. These findings clarify that the partial protection against muscle atrophy observed at 0.33*g* (Fig. 1, H and I) can primarily be attributable to the preservation of type I myofiber size. This observation highlights the myofiber type–specific responses to gravitational loading and provides a mechanistic link between the attenuation of muscle atrophy at 0.33*g* and the incomplete recovery of muscle function, which required 0.67*g* loading.

### Influence of gravity variations in spaceflight on whole gene expression in the SOL muscle

**Fig. 3.**
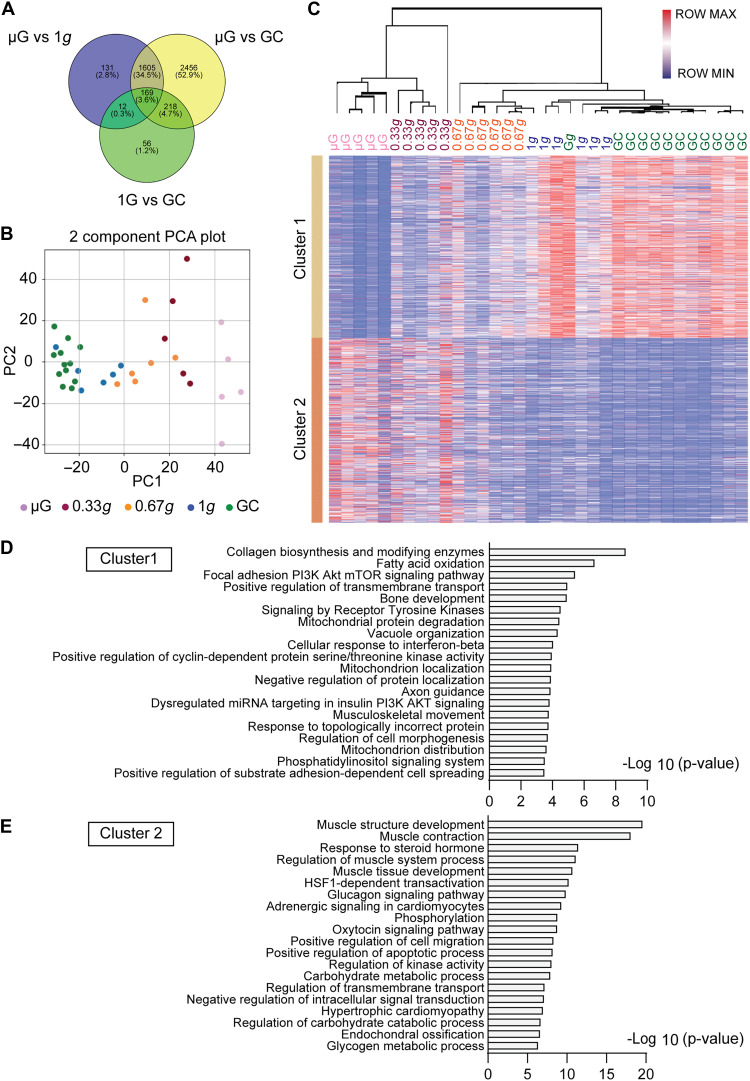
Gravity-dependent changes in global gene expression and pathway enrichment in the SOL during spaceflight. (**A**) Venn diagram of the differentially expressed genes (DEGs) between μG and 1*g*, μG and GC, and 1*g* and GC. (**B**) Two-component PCA plot of 1,605 genes with edgeR false discovery rate (FDR) < 0.05 in the SOL. (**C**) Cluster heat map of 1605 genes with edgeR FDR < 0.05 in the SOL. (**D** and **E**) Pathway analysis using Metascape for clusters 1 (774 genes) and 2 (831 genes). Sample sizes: μG (*n* = 5), 0.33*g* (*n* = 5), 0.67*g* (*n* = 6), 1*g* (*n* = 6), and GC (*n* = 12).

To elucidate the effects of different gravity levels on the whole transcriptome of the SOL, RNA sequencing (RNA-seq) analysis was performed. We identified gravity-responsive genes by comparing the μG group with both the 1*g* and GC groups. To minimize the influence of spaceflight-related factors other than gravity, we excluded genes that differed between the 1*g* and GC conditions. This approach allowed us to enrich a more precise set of genes (1605 genes) whose expression patterns were consistent with regulation by gravitational difference (Fig. 3A). Principal components analysis (PCA) revealed clear sample separation among the groups. Clusters were arranged along the gravity gradient, progressing from μG to 0.33*g*, 0.67*g*, 1*g*, and GC (Fig. 3B). Visualizing differentially expressed genes using a heatmap revealed two distinct gene clusters (Fig. 3C). Cluster 1 consisted of genes that were down-regulated under both μG and 0.33*g* conditions. These genes were enriched in the PI3K-Akt-mTOR signaling pathway, fatty acid oxidation, and mitochondrial localization (Fig. 3D), suggesting that exposure to μG and 0.33*g* suppresses protein synthesis and shifts metabolic demand from oxidative phosphorylation toward glycolytic processes. Cluster 2, characterized by up-regulation under μG and 0.33*g* conditions, comprised genes associated with responses to muscle structure development, steroid hormones, glucagon, and glycogen metabolism. These changes indicate activation of muscle atrophy pathways and a shift in metabolic features from slow- to fast-twitch myofibers, which is consistent with the observed phenotypes of the SOL under μG and 0.33*g* conditions (Fig. 3E).

### Gravity-dependent changes in atrogene and myofiber type–related gene expression

**Fig. 4.**
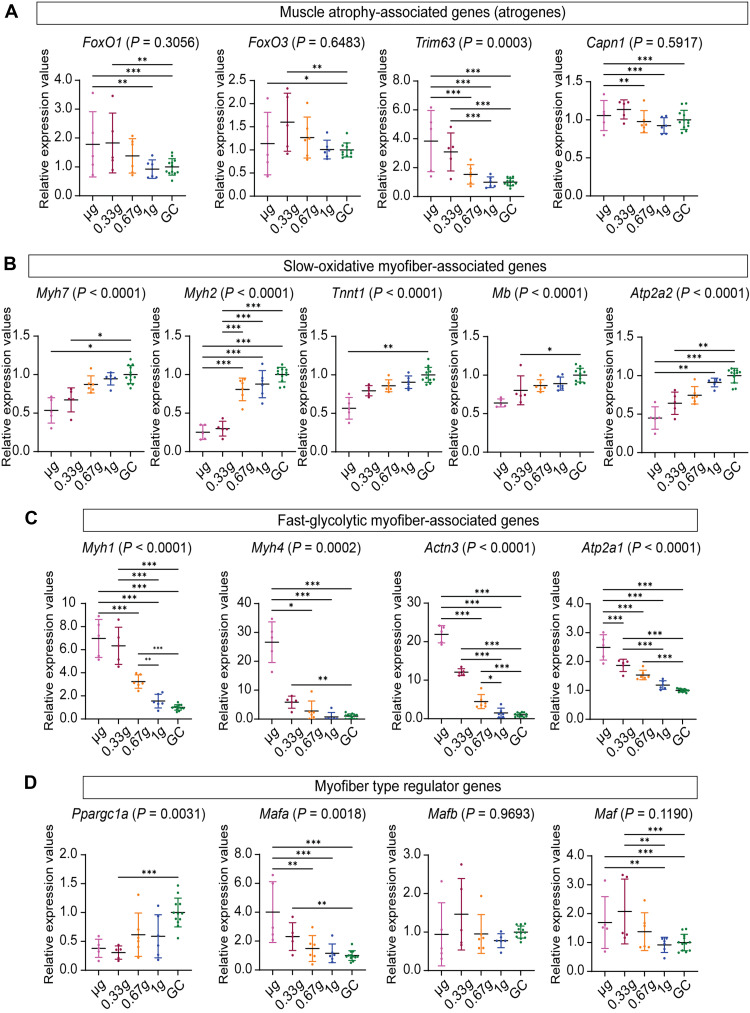
Gravity-dependent changes in expression of genes related to muscle atrophy and myofiber type transition in the SOL during spaceflight. (**A**) Muscle atrophy-associated gene (Atrogene) expression (*Foxo1*, *Foxo3*, *Trim63*, *Capn1*, and *Capn3*) in the SOL. (**B**) Slow-oxidative myofiber-associate genes expression (*Myh7*, *Myh2*, *Tnnt1*, *Mb*, and *Atp2a2*) in the SOL. (**C**) Slow-oxidative myofiber-associate genes expression (*Myh1*, *Myh4*, *Actn3*, and *Atp2a1*) in the SOL. (**D**) Myofiber type regulator gene expression (*Ppargc1a*, *Mafa*, *Mafb*, and *Maf*) in the SOL. Data are shown as mean ± SD. Expression values are normalized by scaling (each GC = 1). Sample sizes: μG: *n* = 5, 0.33*g*: *n* = 5, 0.67*g*: *n* = 6, 1*g*: *n* = 6, and GC: *n* = 12. FDR *P* values were calculated using edgeR: *P < 0.05, **P < 0.01, and ***P < 0.001. FDR-adjusted ANOVA *P* values from the main statistical analysis are shown in parentheses next to each gene name. Post hoc pairwise comparisons are indicated by asterisks.

We further investigated the expression of atrogenes (*Foxo1*, *Foxo3*, *Trim63*, and *Capn1*) known to be induced in various muscle atrophy models (Fig. 4A). *Foxo1*, *Foxo3*, *Trim63*, and *Capn1* expression levels exhibited significant up-regulation in μG and/or 0.33*g* groups compared with that in the 1*g* and/or GC groups (Fig. 4A). Hence, exposure to a gravitational force of 0.67*g* evidently suffices to prevent the up-regulation of atrogene expression during spaceflight. Nevertheless, given that a gravitational level of 0.33*g* partially mitigated the SOL atrophy at a histological level (Fig. 1), it is conceivable that undefined atrogenes may contribute to the atrophic response induced by μG.

In addition, we analyzed genes associated with specific myofiber types (Fig. 4, B and C). Notably, the expression levels of *Myh7* (type I) and *Myh2* (type IIa) increased in a gravity-dependent manner (Fig. 4B). In contrast, the levels of *Myh1* (type IIx) and *Myh4* (type IIb) showed a gravity-dependent decrease (Fig. 4C). These results underscore the slow-to-fast myofiber type transition observed in the immunohistochemical analysis. *Mb* and *Tnnt1*, preferentially expressed in slow-twitch myofibers, exhibited decreased expression levels as gravity decreased (Fig. 4B). Conversely, the expression of *Actn3*, which is abundant in fast-twitch myofibers, markedly increased in a gravity-dependent manner (Fig. 4C). The expression levels of adenosine triphosphatase sarcoplasmic/endoplasmic reticulum Ca2^+^ transporting 1/2 (*Atp2a1* and *Atp2a2*), which are important calcium pumps in fast-twitch and slow-twitch myofibers, respectively, were also examined. As gravity decreased, *Atp2a1* expression increased, while *Atp2a2* expression decreased, both results corresponding with slow-to-fast myofiber type transition in the SOL (Fig. 4, B and C). *Ppargc1a*, which regulates mitochondrial biogenesis and is known as a major factor in determining myofiber type, exhibited decreased expression patterns as gravity decreased, also consistent with metabolic changes in slow-to-fast myofiber type transition in the SOL (Fig. 4D). Notably, the large Maf family transcriptional factors *Mafa* and *Maf*, which were identified to play a major role in type IIb myofiber specification, were significantly up-regulated at μG and/or 0.33*g* but remained unchanged in 0.67*g* compared with that in 1*g*. However, *Mafb* did not show significant alterations at different gravity levels (Fig. 4D). Together, these results show that a gravitational force of 0.67*g* significantly inhibits the activation of genes linked to muscle atrophy and slow-to-fast myofiber type transition in the SOL during spaceflight.

### Metabolomic profiling of plasma under variable gravity

To further explore the systemic physiological responses associated with the observed muscle phenotypes, namely, the preservation of muscle mass at 0.33*g* and the requirement of 0.67*g* to maintain muscle strength, we next performed plasma metabolomic profiling. Our aim was to identify circulating metabolites that reflect muscle responsiveness to altered gravity and potentially serve as biomarkers to predict muscle atrophy or functional decline under μG conditions.

Nuclear magnetic resonance (NMR)–based metabolomic profiling detected 43 plasma metabolites. Hierarchical clustering and PCA followed by k-means clustering showed that the μG, 0.33*g*, and 0.67*g* groups segregated from both the 1*g* and GC groups, indicating distinct metabolic responses to reduced gravitational loading (fig. S4, A and B). However, four mice from the 0.33*g* and 0.67*g* groups formed a separate cluster characterized by markedly elevated ketone bodies (3-hydroxybutyrate, acetoacetate, and acetone) together with reduced amino acids (fig. S4, B and C). This suggested that these four mice were more likely to be in a prolonged fasting state before blood collection, as ketone body elevation and amino acid reduction are well-established hallmarks of fasting metabolism (*23*). To avoid confounding, these four outlier mice were excluded from subsequent analyses.

**Fig. 5.**
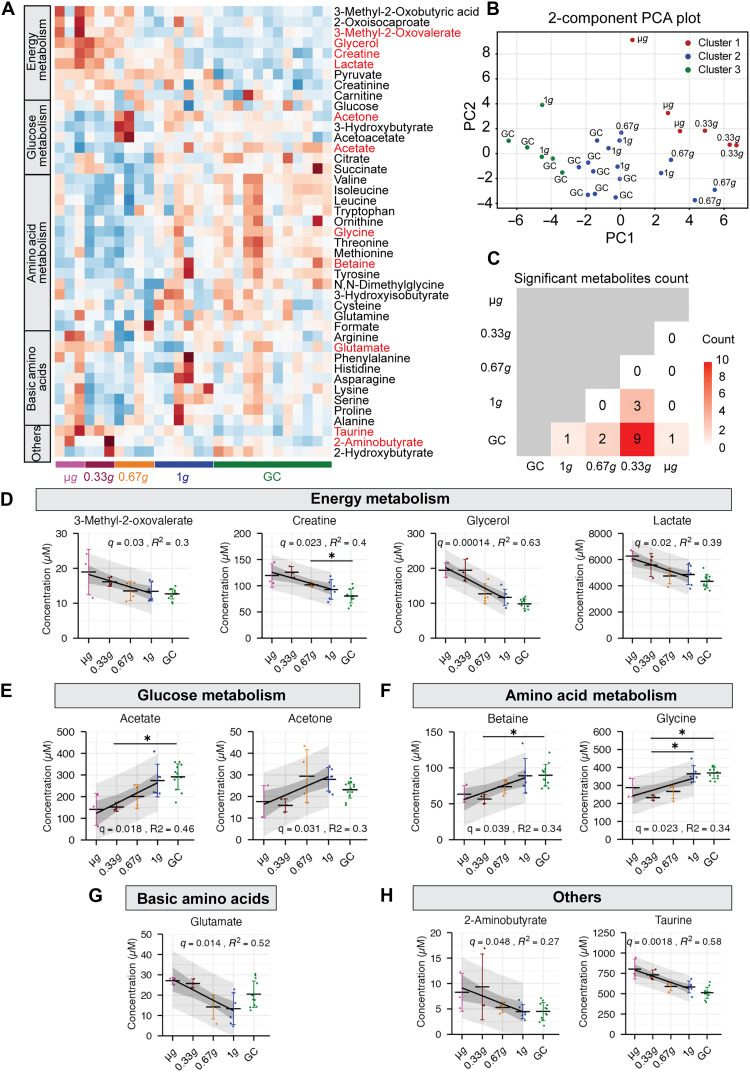
Gravity-dependent changes in circulating metabolites during spaceflight. (**A**) A heatmap of 43 metabolites after excluding four mice showing abnormal elevation of ketone bodies. *Z*-score normalization across all samples (mean = 0 and SD = 1) was applied to each metabolite. Red labels indicate metabolites significant in the subsequent linear regression analysis. (**B**) Two-component principal components analysis (PCA) plot of the 43 metabolites. K-means clustering was performed using the PCs until the cumulative variance explained by the loadings exceeded 80%. (**C**) Pairwise comparison table between each group showing the number of metabolites with significant differences in their concentrations (*q* values <0.05). (**D** to **H**) Concentrations of metabolites showing significant gravity-dependent changes, with linear regression lines indicating the trend across gravity conditions with 95% confidence intervals (darker shading) and 95% prediction intervals (lighter shading). The *q* values were obtained using Student’s *t* test with false discovery rate (FDR) correction applied across all metabolites. *R*^2^ values indicate the proportion of variance explained by gravity level. Data are shown as mean ± SD. Sample sizes: μG: *n* = 3, 0.33*g*: *n* = 3, 0.67*g*: *n* = 4, 1*g*: *n* = 6, and GC: *n* = 12.

After exclusion, a heatmap demonstrated gravity-dependent shifts, particularly in metabolites associated with energy, glucose, and amino acid metabolism (Fig. 5A). PCA further revealed clustering along the gravity gradient, with clear separation between the 0.33*g* and 0.67*g* groups (Fig. 5B). Pairwise group comparisons identified multiple significantly altered metabolites related to energy, glucose, and amino acid metabolism in the reduced-gravity groups compared with that in 1*g* or GC groups (Fig. 5C and data S1). Linear regression analysis using gravity as a continuous variable identified 11 metabolites with a significant gravity-dependent trend (Fig. 5, D to H). Notably, creatine, lactate, glycerol, and glutamate levels were increased, while the levels of several amino acid–related metabolites, including glycine and betaine, were decreased under low-gravity conditions. The elevation of lactate and glycerol indicated enhanced glycolytic activity and lipolysis. Overall, these findings portray a gravity-associated remodeling of energy and amino acid metabolism, consistent with the observed effects of gravity on skeletal muscle.

## DISCUSSION

Our previous study demonstrated that artificial 1*g* in space prevents muscle atrophy and slow-to-fast myofiber type transition in the SOL (*7*). However, the specific gravitational threshold that prevents deleterious alteration of the SOL has remained unclear. In the present study, mice were housed on the ISS and exposed to four distinct gravity levels (μG, 0.33*g*, 0.67*g*, and 1*g*) for 27 to 28 days, while a control group (GC) remained on Earth. We showed that skeletal muscle atrophy and slow-to-fast myofiber type transition induced in the SOL under μG were attenuated in a gravity-dependent manner. Specifically, myofiber atrophy was partially inhibited at 0.33*g*, while 0.67*g* attenuated slow-to-fast myofiber type transition and preserved muscle function. This study provides the experimental evidence to define gravity thresholds that maintain skeletal muscle mass and myofiber type and function during spaceflight.

The SOL weight in both the μG and 0.33*g* groups was significantly reduced compared with that in 1*g*. However, the CSA in the 0.33*g* group did not show a significant decrease compared with that in 1*g*. Analysis of CSA by myofiber type revealed that type I myofibers, which normally have a larger CSA than other myofiber types in SOL, were most affected by spaceflight-induced atrophy, whereas type IIa myofibers, which are smaller under normal conditions, showed less CSA reduction. In addition to the histological findings, forelimb grip strength was significantly reduced in both the μG and 0.33*g* groups. Although histological analysis of the forelimb muscles was not performed, the observed decline in forelimb grip strength suggests that μG and partial gravity (0.33*g*) may also affect other muscle groups beyond the SOL. In contrast, hindlimbs grip strength did not differ significantly between pre- and postflight in any gravity condition, despite significant myofiber atrophy in the hindlimb muscles. This discrepancy between histological and functional outcomes suggests differential responses of forelimb and hindlimb musculature to gravitational loading during spaceflight.

*Foxo1*, *Foxo3*, and *Trim63* (*14*, *16*, *17*) were significantly up-regulated under μG and 0.33*g* compared with that under 1*g* and/or GC, and their expression patterns were consistent with the observed reductions in muscle mass and CSA in the SOL. These findings suggest that canonical atrogenes, previously implicated in ground-based models of muscle atrophy, are also activated under reduced gravity conditions during spaceflight. However, prior spaceflight studies using *Trim63* knockout mice have demonstrated that deletion of *Trim63* alone does not fully prevent μG-induced muscle atrophy, suggesting that additional mechanisms are involved (*18*). In the present study, RNA-seq analysis identified a gene cluster (cluster 2) that was up-regulated in μG and 0.33*g* but not in 0.67*g* or 1*g*. This cluster included genes involved in steroid hormone signaling pathways, which may be relevant to muscle atrophy during spaceflight. In addition, several genes related to calcium handling and intracellular cation balance were differentially expressed. Notably, the expression of the mitochondrial fusion proteins Mitofusin 1 and 2 (Mfn1 and Mfn2), which are essential for ER-mitochondrial tethering and calcium transfer (*24*), decreased in a gravity-dependent manner (fig. S5, A and B). These molecular changes may have contributed to impaired calcium homeostasis in skeletal muscle under μG and 0.33*g*, thereby promoting atrophic processes. Together, these data suggest that gravity-dependent transcriptional responses involve both classical and previously underappreciated pathways, and that the present RNA-seq dataset may serve as a useful resource for identifying additional atrogenes and molecular mediators of muscle atrophy during spaceflight.

In the SOL, the proportion of type IIb myofibers was significantly increased under μG compared with that under 1*g* and GC, while the proportion of type IIa myofibers was significantly decreased under both μG and 0.33*g* compared with that under 1*g* and GC. In contrast, the proportion of type IIx myofibers was significantly higher at 0.33*g* and 0.67*g* than at μG. These results suggest that myofiber type transition in the SOL occurs in a stepwise manner between adjacent myofiber types (IIa ⇔ IIx ⇔ IIb), with the extent of transition dependent on the level of gravitational unloading. Under μG, the transition appears to progress from type IIa through IIx to IIb, whereas under 0.33*g*, the transition may be limited to a shift from type IIa to IIx. Alternately, the proportion of type I myofibers remained unchanged across all gravity conditions, despite clear evidence of myofiber atrophy. This suggests that myofiber atrophy and myofiber type transition occur in distinct myofiber populations within the same muscle, namely, that type I myofibers undergo atrophy, while type IIa and IIx myofibers undergo a slow-to-fast myofiber type transition.

Previously, we identified the large Maf transcription factors (*Mafa*, *Mafb*, and *Maf*) as key regulators of type IIb myofiber specification, with their genetic ablation resulting in an almost complete loss of type IIb myofibers throughout skeletal muscle in mice (*8*). Yet, in the same study, overexpression of *Mafa*, *Mafb*, or *Maf* in the SOL was sufficient to induce type IIb myofiber formation in adult mice. In the present study, the RNA-seq data revealed that *Mafa* and *Maf* were up-regulated in the SOL at μG and 0.33*g*, but not at 0.67*g*. In addition, the expression of *Cacna1s* and *Ryr1*, genes involved in calcium dynamics and known to influence *Maf* expression (*25*), also increased with reduced gravity (fig. S5, C and D). These findings support the existence of a gravity-sensitive Cacna1s-Maf axis in which altered Ca^2+^ dynamics under reduced gravity may drive *Mafa* and *Maf* expression. Beyond its role in fast myofiber specification, *Maf* overexpression has been shown to counter denervation-associated muscle loss by repressing atrogenes such as *Fbxo32* and *Trim63* (*26*). Together, these findings highlight the central role of the large Maf transcription factors in regulating both myofiber type formation and muscle mass, suggesting their potential importance in future spaceflight studies and space-related biomedical applications.

Muscle atrophy, myofiber type transition, and declines in muscle function, such as grip strength and electrical permittivity, were shown to be suppressed at different gravitational thresholds during spaceflight. In this context, changes in circulating metabolites may serve as important biomarkers for capturing physiological alterations in skeletal muscle and other tissues during spaceflight. Eleven metabolites exhibited gravity-dependent increases or decreases (Fig. 5, D to H), suggesting the potential to assess muscle changes without invasive intervention into muscle tissue. These metabolites are thought to exert biological effects that may be functionally related to the skeletal muscle changes observed in this study, although their levels in plasma may also reflect metabolic alterations in other tissues throughout the body.

The PCA of the metabolites highlighted that the samples were distributed according to the gravity gradient, with a clear separation between the 0.33*g* and 0.67*g* groups. This finding suggests that relatively small differences in gravitational loading are sufficient to induce measurable shifts in systemic metabolism. Note that four mice were excluded from this analysis, as they exhibited an abnormal metabolic profile characterized by elevated ketone bodies and reduced amino acid levels (fig. S5C), similar to what has been described in studies of fasting in rodents and humans. Starvation promotes lipolysis, and the breakdown of fat becomes the major source of energy, leading to the production of ketone bodies from β-oxidation (*23*). A recent study demonstrated that ketone body levels began to increase after 6 hours of fasting, accompanied by impaired glucose tolerance in mice (*27*). Thus, our metabolomic data strongly supported the hypothesis that the four mice had undergone a longer fasting period before sample collection. Although food was available in the cages both during and after the return to Earth, some may have experienced difficulty in resuming feeding following their exposure to the high gravitational forces during re-entry. Therefore, these four mice were excluded from the PCA and from subsequent analyses.

The observed increases in creatine, lactate, glycerol, and glutamate under reduced gravity suggest marked alterations in systemic and muscle-related energy metabolism. The increase in lactate could be indicative of a metabolic shift toward glycolysis, while the rise in creatine may reflect either enhanced muscle breakdown or reduced muscular uptake. Creatine has been investigated as a supplement for counteracting muscle atrophy (*28*) and for improving exercise performance (*29*). Therefore, the observed increase in creatine under low-gravity conditions may suggest increased susceptibility to muscle atrophy. Regarding lactate, although it is produced in skeletal muscle as a product of glycolysis, its systemic concentration reflects the balance between muscle production, mitochondrial oxidation, and hepatic clearance via the Cori cycle. The transcriptomic enrichment of glycogen metabolic pathways under low-gravity conditions (Fig. 3E), together with previous findings showing increased lactate accumulation in skeletal muscle during unloading (*30*), suggests that low-gravity conditions may shift skeletal muscle metabolism toward reliance on glycolysis. The stepwise increase in glycerol concentrations with decreasing gravity shown in our analysis likely reflects enhanced lipid mobilization, potentially from adipose tissue, pointing to systemic metabolic adaptations beyond skeletal muscle. After excluding fasting outliers, ketone bodies, including 3-hydroxybutyrate, acetoacetate, and acetone, were found to be reduced under 0.33*g*, 0.67*g*, and μG conditions. Their reduction under low gravity may reflect a preferential reliance on glycolytic metabolism over fatty acid oxidation. Together, these findings highlight the remodeling of systemic metabolism as gravity decreases, characterized by increased markers of glycolysis and lipolysis along with reduced ketone body production.

In parallel, the reductions in betaine and glycine under low gravity conditions suggest a possible decline in systemic antioxidant and anti-inflammatory capacity (Fig. 5D). Glycine depletion could impair glutathione synthesis and redox homeostasis (*31*). In addition, supplementation with GlyNAC, a combination of glycine and *N*-acetylcysteine, has been shown to improve aging hallmarks in humans (*32*), and glycine enhances muscle mass in pigs (*33*). Therefore, the reduction of plasma glycine levels under low-gravity conditions may be consistent with a diminished capacity to resist muscle atrophy during spaceflight. The observed betaine reduction under low gravity conditions is notable given its known role in maintaining cellular antioxidant defense (*34*). This raises the possibility that reduced plasma betaine availability under low-gravity conditions may contribute to increased susceptibility to muscle atrophy during spaceflight. In line with the reproducibility observed in previous Mouse Habitat Unit (MHU) projects, the decrease in glycine and increase in glycerol in the current study mirrored the findings from MHU-3, which were also associated with human aging in a human cohort study (*35*). Plasma metabolomic profiling identified candidate circulating biomarkers for noninvasive monitoring of physiological changes during spaceflight or unloading. These findings indicate that reduced gravity induces systemic metabolic shifts, characterized by increased markers of energy metabolism and lipid mobilization and decreased amino acid metabolism. However, because plasma metabolites reflect contributions from multiple tissues, their specificity for changes in skeletal muscle remains uncertain, and the limited gravity conditions and time points examined warrant further investigation.

The impact of spaceflight is not limited to skeletal muscle alone but also extends to the neuromuscular junction (*36*, *37*) and other cellular functions. The decline of muscle functions observed in the present study may, therefore, result from impaired muscle function and abnormalities in muscle, other organ cross-talk. Because of the limited amount of tissue available from each mouse, we performed only bulk RNA-seq analysis. Thus, the observed transcriptomic and functional changes cannot be definitively attributed to specific cell populations within skeletal muscle (*38*–*41*). Future studies incorporating cell type–specific approaches, such as single-cell/nucleus RNA-seq or in situ RNA-seq, will be essential to clarifying the contributions of distinct cell types to muscle adaptation and dysfunction during spaceflight. Considering other cells that contribute to skeletal muscle characteristics, careful interpretation of the EIM results is also required. EIM is regarded as a reliable assessment method for neuromuscular and muscular disorders, such as amyotrophic lateral sclerosis (*42*) and Duchenne muscular dystrophy (*43*). However, because EIM measures the surface voltage generated when the electric current is applied via electrodes placed on the target muscle, the values may be influenced by abnormalities or changes in nonmuscle tissues and physiological factors, such as the residual water in skeletal muscles, which can alter conductivity. Therefore, it is important to consider these potential influences.

Because of the nature of space experiments, this study has some limitations. First, in our previous space mission, mice were typically dissected ~48 hours after returning to Earth, which could affect several phenotypes, including gene expression (*6*). In the present study, dissections were performed ~6 hours after landing, enabling a more accurate assessment of gravity-induced changes than that in previous study. Nevertheless, although the postlanding dissection time was substantially shortened, we cannot completely exclude the possibility that this interval influenced gene expression and metabolomic profiles. To more precisely capture in-orbit phenotypes, future experiments should aim to preserve and freeze samples onboard the spacecraft, an approach currently being implemented in our group. Second, the small sample size limited our ability to evaluate age- or sex-dependent effects, and it reduced statistical power for some analyses, including metabolomic profiling. Several metabolites exhibited values falling outside the 95% prediction intervals of the linear regression models, which may have reflected limited sample size, substantial inter-individual variability, or potential nonlinear relationships between gravity levels and metabolite concentrations. In addition, the mice used in this study were young adults, whereas astronauts typically span a broader age range and are often middle-aged. We chose to use young adult mice because it minimized the confounding effects associated with growth or age-related alterations and thus allowed for clearer detection of gravity-dependent biological responses. This age range is commonly used in spaceflight and skeletal muscle research for similar reasons. Nevertheless, the age should be considered when comparing our results to those of other studies, or when translating the results to human spaceflight. Third, previous work using a tail-suspension mouse model has shown that intermittent loading alone is often insufficient to fully prevent muscle atrophy (*44*). Our study suggests that continuous partial gravity at 0.67*g* may overcome this limitation in mice, but the exact gravitational thresholds are likely to differ between species because of differences in locomotion and muscle loading patterns, particularly the fundamental distinction between human bipedal and mouse quadrupedal locomotion. Finally, we were unable to analyze multiple time points. Thus, while 0.67*g* was sufficient to prevent major muscle phenotypes, including loss of mass, myofiber type transition, and functional decline, over the short term, its efficacy for longer missions remains uncertain. Considering that a journey to Mars would require approximately 8 to 9 months, it is essential to assess whether the protective effects observed here will persist during extended spaceflight.

In conclusion, this study demonstrated that skeletal muscle atrophy, slow-to-fast myofiber type transition, and functional decline induced by μG are attenuated in a gravity-dependent manner, with distinct gravitational thresholds required for each phenotype. Furthermore, we identified 11 circulating metabolites that exhibited gravity-dependent changes, providing potential noninvasive biomarkers for monitoring muscle and systemic physiological alterations during spaceflight. These findings not only define the gravitational thresholds necessary to maintain skeletal muscle health in space but also offer valuable resources for future biomarker development to support long-duration human space missions. Artificial gravity environments in space could serve as an alternative to the several hours of daily exercise required of astronauts, offering a practical countermeasure that protects skeletal muscle function during long-duration missions.

## MATERIALS AND METHODS

### Animals

C57BL/6J male mice (stock no. 000664) were purchased from Jackson Laboratories (Bar Harbor, ME, USA) and subcutaneously implanted with radio-frequency identification chips (IMI-500; Bio Medic Data Systems, Seaford, DE, USA) by the vendor before shipment. All experiments were approved by the Institutional Animal Care and Use Committees of JAXA (protocol number: 022-008, and NASA; protocol number: FLT-22-154, MHU-8). All experiments were conducted in accordance with the guidelines and applicable laws of Japan and the USA.

During the mission, veterinarians monitored the health of each mouse daily using a video system. The forelimbs and hindlimbs were in contact with the bottom of the habitation cage under 1*g*, 0.67*g*, and 0.33*g* conditions. Health checks during onboard habitation, conducted by the SSPF Science Annex Animal Care Facility Attending Veterinarians, Flight Attending Veterinarians, and the NASA and JAXA Science teams, showed no abnormalities in the eyes, ears, teeth, fur, and tails of the flight mice that would require their removal from the study (*45*).

Mice for the spaceflight experiment were selected based on body weight and the following criteria. Approximately 6-week-old mice were delivered to the Animal Care Facility in the Space Station Processing Facility Science Annex. The first down-selection process excluded mice that exhibited health concerns, suboptimal or poor preflight metrics [<1 fecal pellet, dual-energy x-ray absorptiometry (DXA), gait analysis using DigiGait (Mouse Specifics Inc., Seaford, DE, USA)], aggressive behavior, or abnormally low or high food or water intake. A Mahalanobis distance plot (final body weight versus body weight slope) was used for the second down-selection to identify 115 eligible mice. The final down-selection was based on a principal investigator–specific algorithm. Twenty-four mice (11 weeks old) were selected for the flight experiment and loaded into two transporter cage units (TCUs). Three days after loading, 12 additional mice (11 weeks old) were assigned to the GC group. On that day, they were placed into a ground TCU, exposed to flight simulation in a flight-like habitat environment, and subsequently transferred to the ISS Environmental Simulation Chambers.

A temperature/humidity data logger was attached to the TCU during the launch, onboard, and return phases and inside the Cell Biology Experimental Facility (CBEF) during the onboard phase. The logger data were processed postmission. Carbon dioxide concentration was monitored using a sensor inside the CBEF, and the levels were maintained within an acceptable range throughout the mission. The time course of the changes in environmental parameters is shown in fig. S6 (A to C).

### Flight experiment

MHU-8 flight mice were launched from NASA Kennedy Space Center (KSC) to the ISS in two TCUs aboard a SpaceX Falcon 9 rocket as part of the Space X CRS-27 (Dragon) mission on 15 March 2023. They were housed in the JAXA MARS onboard the ISS’s Kibo module for ~35 days before returning to the Pacific Ocean on 16 April 2023. The mice were subjected to spaceflight conditions and μG (*n* = 6 mice) and artificial gravity (0.33*g*, *n* = 6; 0.67*g*, *n* = 6; and 1*g*, *n* = 6 mice) using the JAXA Habitat Cage Unit and JAXA Kibo centrifuge hardware (CBEF-L; configuration of two artificial gravity compartments; configuration 2 CBEF-ALT or CBEF-Left; https://humans-in-space.jaxa.jp/en/biz-lab/experiment/facility/pm/cbef-l/). Each mouse was housed in one TCU for transportation to and from the ISS and was transferred into an individual Habitat Cage Unit once on the ISS. For MHU-8, the centrifuge configuration had a rotation radius of 0.15 m, consistent with earlier MHU missions (*2*, *7*). The rotation rates for generating 0.33*g*, 0.67*g*, and 1*g* on the ISS were 44, 63, and 77 rpm, respectively, in MARS using a short radius centrifuge (movie S1). The capsule splashed down on 15 April 2023 (GMT). At the time of return, all mice were 15 weeks old; one mouse in the μG group died prior to sample collection. A NASA veterinarian confirmed that the remaining mice exhibited no abnormalities in health or behavior after return. The mice were euthanized 6 hours after landing, after which postflight measurements, including body mass, fecal collection, motor performance, gait analysis, grip strength, and DXA scanning before cardiac blood puncture15, were performed.

### GC experiment

The GC group comprised 12 age-matched (11 weeks old) male C57BL/6J mice, maintained under similar habitat conditions as those of the flight mice exposed to 1*g* within the ISS Environmental Simulation. The operations were conducted with a 3-day delay at KSC. All mice were euthanized the day after their return to Earth.

### NMR-based metabolomic analysis and data quality control

Metabolomic profiling of blood samples collected from the inferior vena cava was performed using NMR spectroscopy, as previously described (*35*, *46*). For this purpose, plasma metabolites were extracted from 50 μl of plasma using a standard methanol extraction method. NMR experiments were performed at 298 K on a Bruker Avance 600-MHz spectrometer (Bruker Corporation, Billerica, MA, USA) equipped with a CryoProbe and a SampleJet sample changer. Consistent with the muscle analysis, one mouse in the μG group was excluded due to death during the space experiment, and one mouse in the 0.33*g* group was excluded because of a >10% body weight loss that was likely attributable to a water nozzle malfunction. For samples in the NMR analysis, one mouse from the μG group was excluded because of insufficient plasma volume and another mouse from the μG group was excluded because the eight metabolites were not quantified due to poor measurement quality, which may be attributed to tissue contamination. For 44 metabolites in the NMR analysis, ethanol data were excluded due to poor measurement quality. After quality control, a total of 43 metabolites from 32 mouse samples (μG: *n* = 3, 0.33*g*: *n* = 5, 0.67*g*: *n* = 6, 1*g*: *n* = 6, and GC: *n* = 12), without missing data, were analyzed. After excluding four mice exhibiting abnormal elevation of ketone bodies, we lastly analyzed 28 mouse samples (μG: *n* = 3, 0.33*g*: *n* = 3, 0.67*g*: *n* = 4, 1*g*: *n* = 6, and GC: *n* = 12). For each metabolite, pairwise group comparisons were conducted using Student’s *t* tests. To control for multiple testing, *q* values were calculated by applying the Benjamini-Hochberg false discovery rate (FDR) procedure across all pairwise comparisons of all metabolites (10 comparisons × 43 metabolites = 430 tests). Results with *q* < 0.05 were considered statistically significant.

To evaluate the dose-response effects of gravity on metabolite concentrations, we performed a linear regression analysis for each metabolite using the following model: Metabolite concentration ~β_0_ + β_1_ × Gravity, where Gravity represents the gravity level as a continuous variable (0 for μG, 0.33 for 0.33*g*, 0.67 for 0.67*g*, and 1.0 for 1*g*). For each metabolite, regression coefficients were tested for significance using *t* tests, with FDR correction applied across all metabolites. The coefficient of determination (*R*^2^) was calculated to assess the proportion of variance in metabolite concentration explained by gravity level. To visualize the uncertainty associated with the regression analysis, we calculated both 95% confidence intervals and 95% prediction intervals using the predict function in R. The 95% confidence interval represents the range within which the true mean response is expected to fall with 95% probability, reflecting uncertainty in the position of the regression line. The 95% prediction interval represents the range within which a new individual observation is expected to fall with 95% probability.

### Grip strength testing

Mouse grip strength was tested before and immediately following live animal return using a 50-N capacity grip strength meter (Conduct Science, Skokie, IL, USA). The person performing the grip strength testing was blinded to the experimental conditions during flight. We also measured grip strength in GC animals set on a 2-day delay to match the flight environmental conditions. For front paw testing, the mice were lowered over a bar until they gripped it. The experimenter gently pulled the base of the tail to ensure that the animal remained horizontal and then continued pulling until the mouse released its grip. For hind paw testing, the mice were gently restrained, and their hind paws were positioned near the bar until they gripped it. The experimenter gently pulled the mouse until it released its grip. Three trials were performed for both tests, and the mean peak force of the trials was used for subsequent analyses.

### Ex vivo EIM

EIM was performed using an mView System (Myolex Inc., Boston, MA, USA). Ex vivo EIM was performed on the excised left GAS, which was sectioned to fit into a dielectric cell (5 mm by 5 mm), as previously described (*47*). Reference values were obtained by measuring saline solution (0.9% NaCl) before the experiments, and GAS height was recorded. Measurements were obtained at 41 frequencies (1 kHz to 10 MHz) in the transverse direction (i.e., current flowing perpendicular to the major myofiber orientation). We analyzed the values of these parameters at 50 and 500 kHz. A higher frequency reflects contributions from intra- and extracellular compartments. Intrinsic electrical muscle properties (conductivity and relative permittivity) were calculated using saline data for normalization, as previously described (*48*, *49*).

### Sample collection and preparation

The left and right SOL and EDL were stacked neatly on top of each other. The Achilles tendon side was vertically mounted on tragacanth gum on a cork disc and quickly frozen in isopentane cooled in liquid nitrogen. Thin-frozen muscle sections (8 μm) were subjected to immunohistochemical and RNA-seq analyses.

### RNA-seq analysis

RNA was extracted from 100 8-μm SOL tissue frozen sections using TRIzol (Thermo Fisher Scientific, Waltham, MA, USA). An RNA-seq library was prepared using a NEBNext Ultra II Directional RNA Library Prep Kit (New England Biolabs, Ipswich, MA, USA) after ribosomal RNA (rRNA) depletion using a NEBNext rRNA depletion kit (New England Biolabs). Paired-end (2 × 36 bases) sequencing was performed using a NextSeq500 platform (Illumina, San Diego, CA, USA). The generated FASTQ files were imported into CLC Genomics Workbench (version 10.1.1; Qiagen, Hilden, Germany) for further analysis. The sequence reads were mapped to the mouse reference genome (mm10). Gene expression was quantified by calculating the total read count normalized to transcripts per million. Genes with zero counts in any sample were excluded. Differential expression analysis was conducted using edgeR empirical analysis. Differentially expressed genes common to μG versus 1*g* and μG versus GC were identified, whereas those common to 1*g* versus GC were excluded, with an edgeR FDR-corrected *P* value less than 0.05. A PCA plot was constructed using Python to visualize the data. The RNA-seq data were deposited in the DNA Databank of Japan (DDBJ) under accession number BioProject PRJDB17587.

### Gene functional analysis

A clustering heatmap was generated using Morpheus software (https://software.broadinstitute.org/morpheus/). The Gene Ontology analysis of each cluster was performed using the Metascape web tool (https://metascape.org/gp/index.html).

### Histological and immunohistochemical analyses of muscle cryosections

Eight-micrometer frozen sections of the SOL and EDL were mounted on glass slides and subjected to H&E staining and immunohistochemical analysis. For H&E staining, the sections were air-dried and washed with water for 3 min. Subsequently, the sections were nuclear stained with a hematoxylin solution for 8 min, washed with water for 3 min, and eosin stained using a cytoplasmic solution for 1 min. After staining, the sections were dehydrated with 95% ethanol for 5 s twice and 100% ethanol for 5 s and penetrated with xylene for 5 min thrice. Tissue sections were subsequently fixed with dibutyl phthalate polystyrene xylene. For immunohistochemical analysis, the sections were air-dried, fixed at −20°C with acetone for 10 min, air-dried in a wet box for 20 min, and blocked with 5% goat serum/1% bovine serum albumin/phosphate-buffered saline (PBS) and M.O.M. blocking reagent (Vector Laboratories, Newark, CA, USA) for 1 hour at 20° to 25°C. Thereafter, the sections were incubated with primary antibodies and M.O.M. protein concentrate (Vector Laboratories) overnight at 4°C. The primary mouse monoclonal antibodies [Developmental Studies Hybridoma Bank (University of Iowa, Iowa City, IA, USA)] BA-D5 (1:50), SC-71 (1:100), and BF-F8 (1:100) were used against Myh7 for type I myofibers, Myh2 for type IIa myofibers, and Myh4 for type IIb myofibers, respectively. After incubation with the primary antibodies, all samples were washed with PBS and incubated with the appropriate Alexa Fluor–conjugated secondary antibody (Thermo Fisher Scientific, 1:1000) for 1 h at room temperature. The tissue sections were subsequently affixed using VECTASHIELD Vibrance Antifade Mounting Medium (Vector Laboratories) for 30 min after washing with PBS.

The distribution of the CSA of the myofibers and each myofiber type was evaluated using a BIOREVO BZ-X800 microscope system, along with a hybrid cell counting application (Keyence, Osaka, Japan). A total myofiber count was conducted on samples from the SOL and EDL, comprising 183 to 940 myofibers per section. Myofiber types with a count of 0 were assumed to be N.D. and are not represented as dots on the graph.

### Statistics and reproducibility

All data are presented as the means of biological replicates ± SD. Individual sample data are graphically represented by dots using GraphPad Prism (GraphPad Software Inc., San Diego, CA). Comparisons between groups were performed using a paired *t* test for body weight and grip strength alterations preflight versus postflight. Body and muscle weight comparisons between groups were performed using one-way analysis of variance (ANOVA), followed by Tukey’s test. Statistical significance was set at *P* < 0.05.
