# Supplementary material for: 0.33g mitigates muscle atrophy while 0.67g preserves muscle function and myofiber type composition in mice during spaceflight
Source: Sci Adv. 2026 Mar 13;12(11):eaed2258. doi: 10.1126/sciadv.aed2258 (PMC12985678; doi:10.1126/sciadv.aed2258)
Supplement: Supplementary file 1 — Figs. S1 to S6 Legend for data S1 Legend for movie S1 [file sciadv.aed2258_sm.pdf]

Science Advances
AAAS

Supplementary Materials for

0.33*g* mitigates muscle atrophy while 0.67*g* preserves muscle function and myofiber type composition in mice during spaceflight

Ryosuke Tsuji *et al.*

Corresponding author: Ryo Fujita, fujiryo@md.tsukuba.ac.jp; Dai Shiba, shiba.dai@jaxa.jp;
Satoru Takahashi, satoruta@md.tsukuba.ac.jp

Sci. Adv. **12**, eaed2258 (2026)
DOI: 10.1126/sciadv.aed2258

The PDF file includes:

Figs. S1 to S6
Legend for data S1
Legend for movie S1

Other Supplementary Material for this manuscript includes the following:

Data S1
Movie S1

Supplementary Materials

Supplementary Figure 1 (Tsuji et al.)

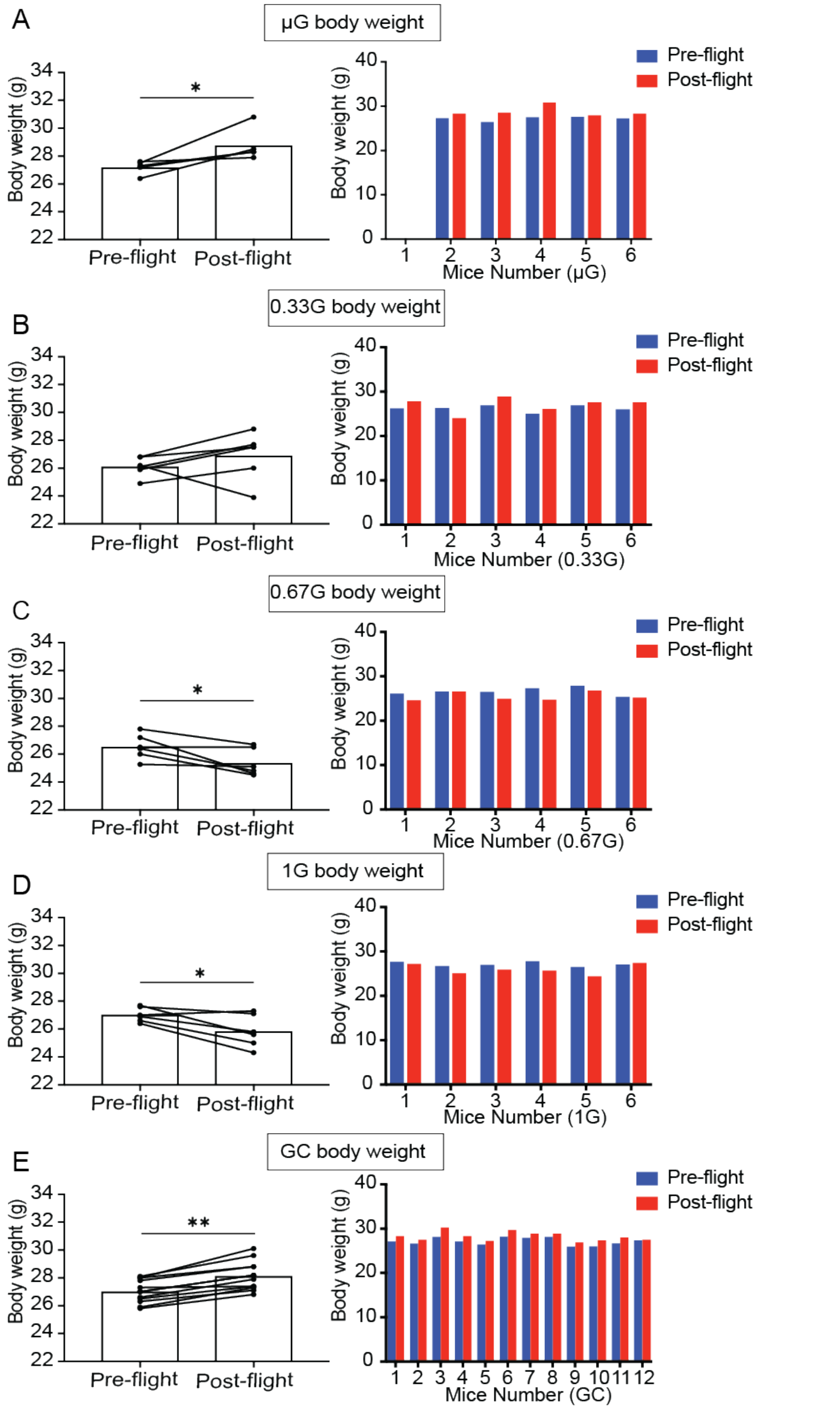

Supplementary Fig. 1. Body weight alteration pre- vs post-flight.

(A) Body weight difference in microgravity (μG) pre- vs. post-flight (left). Body weight alteration of each mouse in μG pre- vs post-flight (right). $n = 5$

(B) Body weight difference at 0.33G pre- vs. post-flight (left). Body weight alteration of each mouse at 0.33G pre- vs. post-flight (right). $n = 6$

(C) Body weight difference at 0.67G pre- vs. post-flight (left). Body weight alteration of each mouse at 0.67G pre- vs post-flight (right). $n = 6$

(D) Body weight difference at 1G pre- vs post-flight (left). Body weight alteration of each mouse at 1G pre- vs. post-flight (right). $n = 6$

(E) Body weight differences between GC pre- and post-flight (left). Body weight alteration in each mouse in GC pre- and post-flight (right). $n = 12$

*$p < 0.05$ and **$p < 0.01$, as determined using a paired *t*-test.

Supplementary Figure 2 (Tsuji et al.)

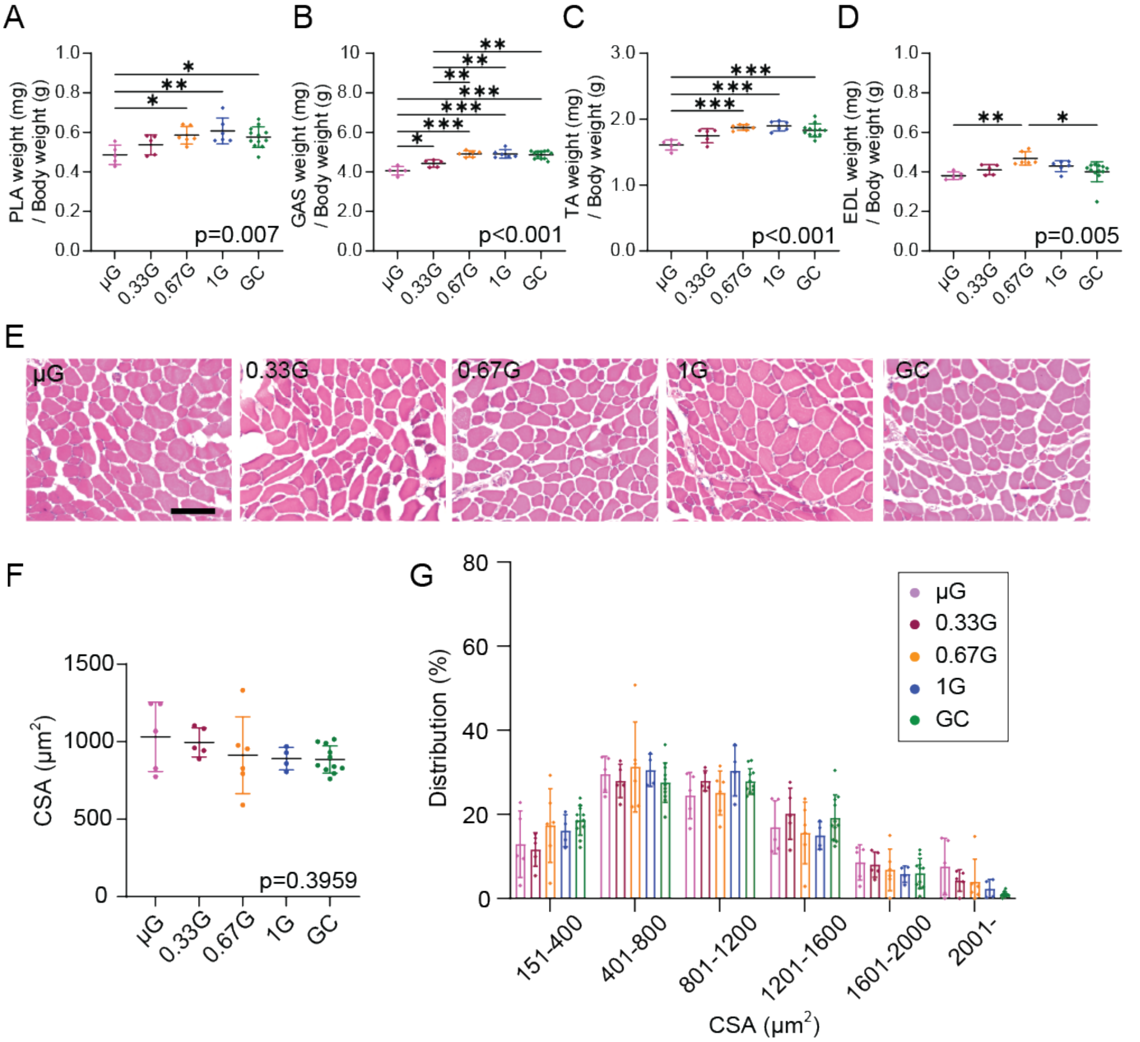

Supplementary Fig. 2. Gravity-dependent hindlimb muscle mass and muscle atrophy in EDL during spaceflight

(A–D) Plantaris (PLA), Gastrocnemius (GAS), Tibialis anterior (TA), and extensor digitorum longus (EDL) muscle weights normalized by body weight. Data are shown as the mean ± SD. μG: n = 5, 0.33G: n = 5, 0.67G: n = 6, 1G: n = 6, GC: n = 12. *$p < 0.05$, **$p < 0.01$, and ***$p < 0.001$, as determined using Tukey's test.

(E) H&E staining of the EDL cross-sections. Scale bar: 100 μm.

(F) Cross-sectional area (CSA) of the EDL myofibers. Data are shown as the mean ± SD. μG: n = 5, 0.33G: n = 5, 0.67G: n = 6, 1G: n = 4, GC: n = 11.

(G) CSA distribution of the EDL myofibers. Data are shown as the mean ± SD. μG: n = 5, 0.33G: n = 5, 0.67G: n = 6, 1G: n = 4, GC: n = 11.

For each quantitative panel, the *p*-value from the main statistical analysis (one-way ANOVA testing the overall effect of gravity level) is shown within the graph. Post hoc pairwise comparisons are indicated by asterisks.

Supplementary Figure 3 (Tsuji et al.)

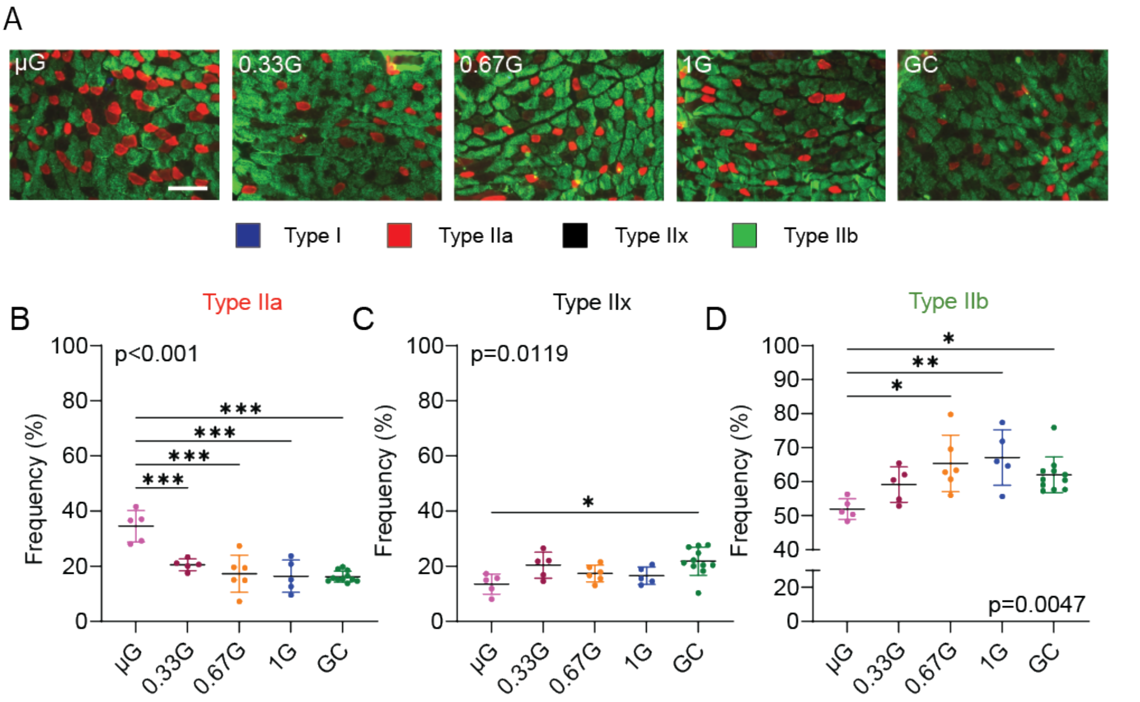

Supplementary Fig. 3. Gravity-dependent changes in myofiber-type shift in the EDL during spaceflight

(A) Immunohistochemical staining of myosin heavy chain using BA-D5 (type I; blue), SC-71 (type IIa; red), and BF-F3 (type IIb; green) antibodies. Unstained myofibers are defined as type IIx (black). Scale bar: 100 μm.

(B-D) Frequency of type IIa/IIx/IIb myofibers in the EDL under different gravity conditions. Data are shown as the mean ± SD. μG: $n = 5$, 0.33G: $n = 5$, 0.67G: $n = 6$, 1G: $n = 5$, GC: $n = 11$. $*p < 0.05$, $**p < 0.01$, and $***p < 0.001$, as determined using Tukey's test.

The frequency of type I myofibers in the EDL, which was not detected under any distinct gravity conditions, was excluded.

For each quantitative panel, the *p*-value from the main statistical analysis (one-way ANOVA testing the overall effect of gravity level) is shown within the graph. Post hoc pairwise comparisons are indicated by asterisks.

Supplementary Figure 4 (Tsuji et al.)

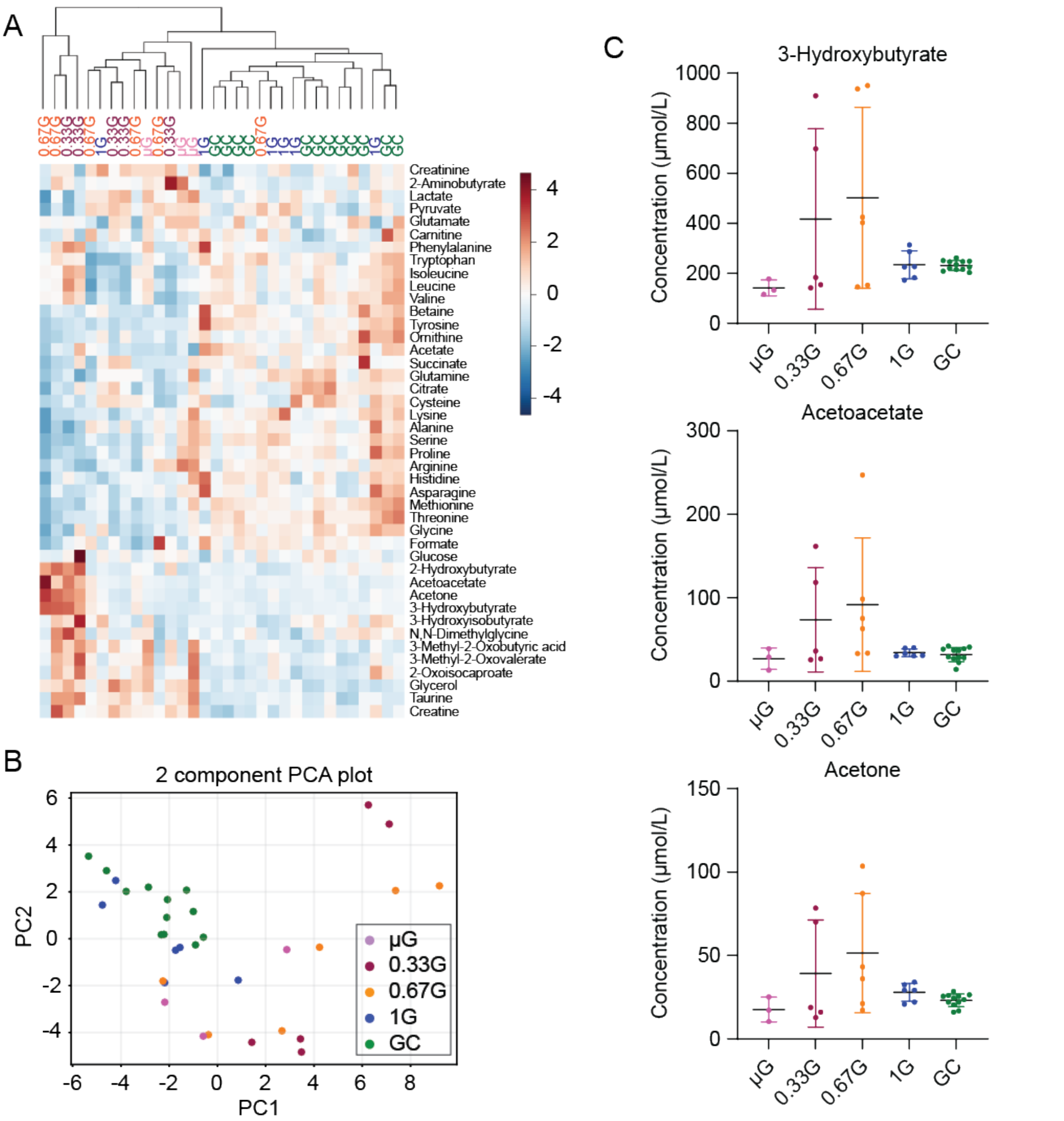

Supplementary Fig. 4. Distinct clustering driven by ketone body outliers in metabolome analysis **(A)** A heatmap of 43 metabolites in NMR-based metabolomic analysis followed by hierarchical clustering (Morpheus). Z-score normalization across all samples (mean = 0, standard deviation = 1) was applied to each metabolite. **(B)** Two-component principal component analysis (PCA) plot of the 43 metabolites. **(C)** Concentrations of ketone bodies (3-hydroxybutyrate, acetoacetate, and acetone), showing abnormal elevations in four mice. Data are shown as mean ± SD. μG: *n* = 3, 0.33G: *n* = 5, 0.67G: *n* = 6, 1G: *n* = 6, GC: *n* = 12.

Supplementary Figure 5 (Tsuji et al.)

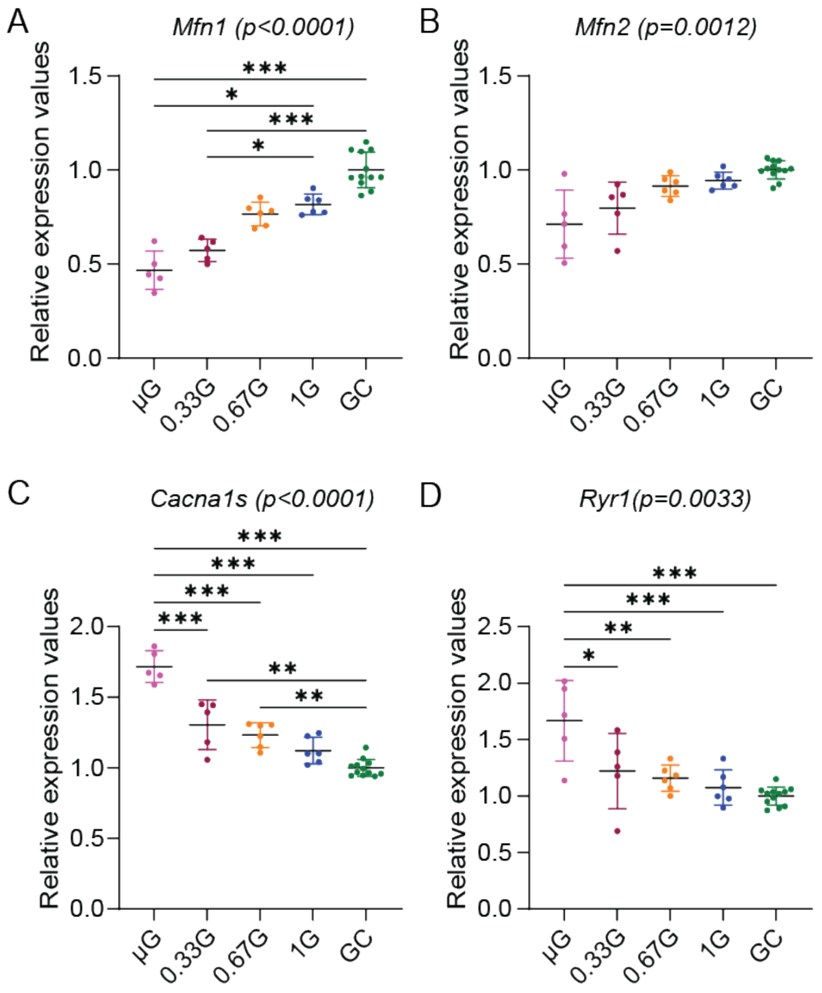

Supplementary Fig. 5. Gravity-dependent changes in the expression of calcium-related genes in the SOL during spaceflight.

(A–D) Gene expression of *Mfn1*, *Mfn2*, *Cacna1s*, and *Ryr1* in the SOL. Data are shown as mean ± SD. Expression values were normalized by scaling (each GC = 1). μG: $n = 5$, 0.33G: $n = 5$, 0.67G: $n = 6$, 1G: $n = 6$, GC: $n = 12$. False discovery rate p-values were calculated using edgeR: $^{*}p < 0.05$, $^{**}p < 0.01$, and $^{***}p < 0.001$.

FDR-adjusted ANOVA p-values from the main statistical analysis are shown in parentheses next to each gene name. Post hoc pairwise comparisons are indicated by asterisks.

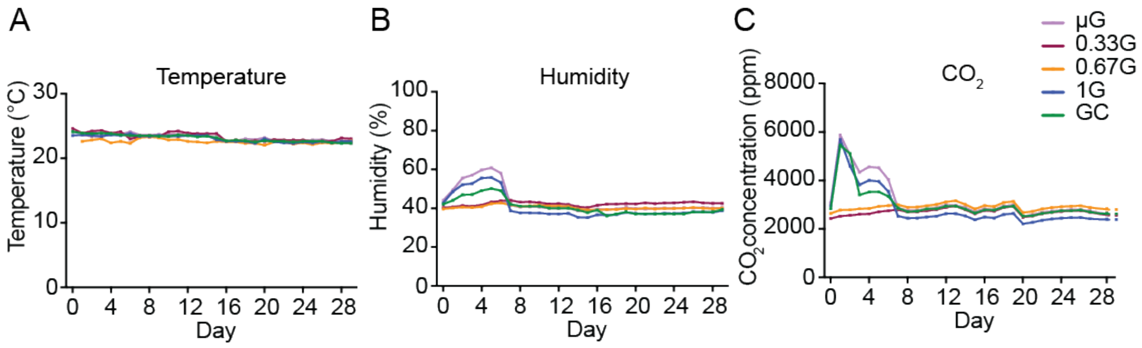

Supplementary Fig. 6. Environmental parameters during the flight mission.

(A) Temperature changes during the mission. **(B)** Humidity changes during the mission. **(C)** Changes in CO_2 during the mission.

Supplementary Data 1. List of circulating metabolites after spaceflight under different gravity conditions

Supplementary Movie 1. Onboard habitation of spaceflight mice at the mission midpoint (Day 16).

Top panel: Spaceflight mice exposed to microgravity; Second panel: Spaceflight mice exposed to 0.33 G; Third panel: Spaceflight mice exposed to 0.67 G; Fourth panel: Spaceflight mice exposed to 1 G; Bottom panel: Ground control mice.
